# A Developmental Perspective on the Intestinal Microbiota in Crohn’s Disease

**DOI:** 10.3390/ijms27052144

**Published:** 2026-02-25

**Authors:** Marcello Imbrizi, Daniela Oliveira Magro, Andrey Santos, Heloisa Balan Assalin, Dioze Guadagnini, Mario José Abdalla Saad, Claudio Saddy Rodrigues Coy

**Affiliations:** 1 Division of Gastroenterology, Department of Internal Medicine, University of Campinas (UNICAMP), 420 Carlos Chagas St, Campinas 13083-878, SP, Brazil; 2 Department of Surgery, University of Campinas (UNICAMP), Campinas 13083-878, SP, Brazil; danimagro06@gmail.com (D.O.M.); claudiocoy@gmail.com (C.S.R.C.); 3 Department of Internal Medicine, School of Medical Sciences, University of Campinas (UNICAMP), Campinas 13083-878, SP, Brazil; andreysts@gmail.com (A.S.); hassalin@unicamp.br (H.B.A.); dioze@unicamp.br (D.G.); msaad@unicamp.br (M.J.A.S.)

**Keywords:** Crohn’s disease, gut microbiome, dysbiosis, inflammatory bowel disease, host–microbiome, barrier function, metabolites, immune regulation, multi-omics

## Abstract

Crohn’s disease (CD) is a chronic inflammatory disorder arising from the convergence of genetic susceptibility, immune dysregulation, environmental exposures, and perturbations of the gut microbiome. This review advances a developmental and compartment-aware framework for interpreting dysbiosis in CD, integrating spatial heterogeneity, transmural pathology, and mesenteric interactions. By synthesizing evidence on microbial composition, functional metabolism, and host-immune crosstalk, we describe a dysbiotic profile shaped by disease location, inflammatory activity, and therapeutic exposure, while also considering the emerging roles of non-bacterial members. We propose that microbiome alterations in CD reflect inflammation-driven ecosystem instability rather than a static taxonomic imbalance. Moving beyond descriptive compositional profiling toward a dynamic ecological model that incorporates disease trajectory and anatomical compartmentalization is essential to refine disease stratification and guide future microbiome-informed precision therapies.

## 1. Introduction

Crohn’s disease (CD) is a subtype of inflammatory bowel disease (IBD), a chronic immune-mediated condition characterized by gastrointestinal inflammation and systemic manifestations [1,2]. Although IBD includes both CD and ulcerative colitis (UC), this review focuses specifically on CD due to its distinct clinical behavior, pathophysiology, transmural inflammatory pattern, and host–microbiome interactions. Rather than reflecting a static dysbiotic state, the microbiota in CD is better conceptualized as an evolving ecological trajectory shaped by host genetics, early-life microbial programming, environmental exposures, and cumulative inflammatory remodeling. This perspective shifts the emphasis from compositional cataloging toward developmental pathobiology [3].

The prevalence of CD varies markedly across geographic regions, with the highest incidence reported in industrialized societies. This distribution has been associated with environmental factors linked to modernization, including urbanization, westernized dietary patterns, reduced microbial exposure, limited sunlight, and antibiotic overuse [4]. These exposures are thought to reshape intestinal microbial ecosystems, reinforcing the concept that environment-driven ecological shifts contribute to CD pathogenesis [5,6]. Understanding the dynamic interplay among environmental pressures, host susceptibility, and microbial ecology is therefore central to both mechanistic insight and therapeutic innovation.

Beyond summarizing microbial composition, this review proposes a developmental and compartment-aware framework for interpreting dysbiosis in Crohn’s disease. We integrate luminal, mucosal, transmural, and mesenteric compartments into host–microbe models and examine how disease heterogeneity, anatomical location, inflammatory activity, and therapeutic exposure shape microbiome findings. By incorporating early-life programming, spatial ecology, transmural pathology, mesenteric adipose interaction, and treatment-induced remodeling, we reposition dysbiosis as a context-dependent ecological process rather than a static taxonomic signature.

## 2. Literature Review Strategy

This article presents a narrative and integrative review of the literature on interactions among the gut microbiome, microbial metabolites, immune regulation, and Crohn’s disease. Relevant studies were identified through structured searches of PubMed/MEDLINE, EMBASE, and Web of Science. The search included articles published from January 2000 through March 2026, with additional cross-referencing of cited articles to ensure comprehensive coverage.

Search strategies combined Medical Subject Headings (MeSH) and free-text terms, including “Crohn’s disease,” “inflammatory bowel disease,” “gut microbiota,” “intestinal microbiome,” “microbial metabolites,” “short-chain fatty acids,” “tryptophan metabolism,” “bile acids,” and “host–microbiome interactions.”

Priority was given to original human studies, translational research, and mechanistic animal models directly relevant to Crohn’s disease. Systematic reviews and high-quality narrative reviews were included to contextualize key findings. Studies not directly related to Crohn’s disease or lacking peer review were excluded. When evidence was heterogeneous, conflicting, or limited, emphasis was placed on studies with clear phenotypic characterization and defined sampling methodology.

Given the marked heterogeneity of disease phenotypes, sampling strategies, and analytical methodologies in Crohn’s disease, a descriptive and mechanistic synthesis was adopted rather than a formal systematic review or meta-analysis.

## 3. The Human Microbiome

**Figure 1:**
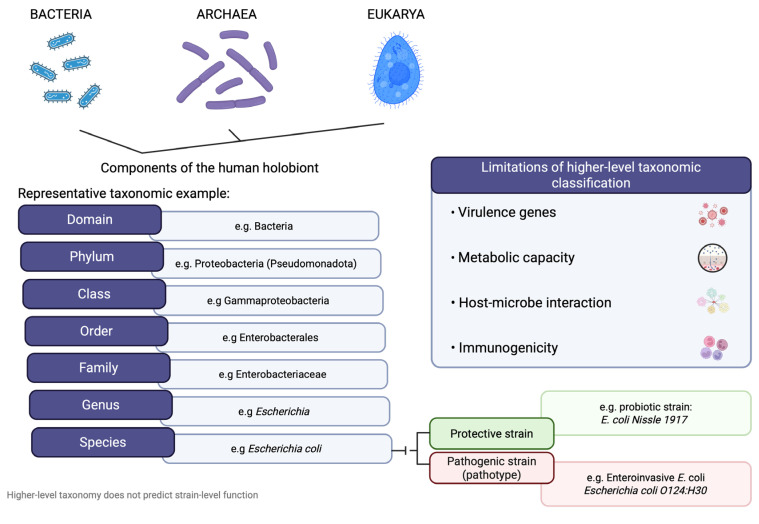
Microorganisms are organized hierarchically from domain to species. Using Escherichia coli as a representative example, the figure illustrates how strain-level variation can result in either protective or pathogenic effects despite identical higher-level taxonomy, highlighting the limitations of taxonomy alone for inferring biological function. Created in BioRender. Saad, M. (2026) https://app.biorender.com/illustrations/695e5afe05e958d3b50d194d.

Bacteria emerged approximately 3.8 billion years ago, long before the appearance of eukaryotes. Since then, many lineages have evolved intimate associations with host organisms. Together with archaea, fungi, protists, helminths, and viruses, they form the holobiont, a biological unit comprising the host and its symbiotic microbial communities [7]. Although the three-domain classification proposed by Carl Woese underpins microbiome taxonomy, most studies report changes at higher taxonomic levels, potentially overlooking functionally relevant strain-level differences in Crohn’s disease. [8,9] Accordingly, microbial taxonomic similarity does not imply functional equivalence. Strain-level variation may confer either protective or pathogenic effects, as exemplified by *Escherichia coli* strains: the probiotic *E. coli* Nissle 1917 and pathogenic enteroinvasive *E. coli* exert divergent immunological and inflammatory effects in the gut (Figure 1) [10,11].

While the Human Genome Project identified approximately 20,000 protein-coding genes, the human microbiome harbors a substantially larger genetic repertoire, with more than 100,000 microbial genes, and constitutes a central component of the holobiont [12,13]. Microbial colonization begins at birth and is shaped by early-life exposures, including mode of delivery, feeding practices, and antibiotic use. These factors critically influence microbiome maturation and long-term immune outcomes, including susceptibility to inflammatory bowel disease [14,15].

After birth, the gut microbiota progressively matures, with increasing alpha diversity and decreasing beta diversity. This transition leads from *Bifidobacterium*-enriched infant communities to a stable adult configuration dominated by *Bacteroidetes* and *Firmicutes*, followed in later life by age-related loss of diversity and expansion of *Proteobacteria* [14,15].

## 4. The Human Gut Microbiome: Ecological Succession, Spatial Organization, and Context-Dependent Dynamics

The intestinal microbiome represents the most extensively characterized human-associated microbial ecosystem due to its remarkable diversity and central role in host physiology. Large-scale initiatives, including the Human Microbiome Project and MetaHIT, have cataloged thousands of gut bacterial species, predominantly within the phyla *Bacteroidetes*, *Firmicutes*, *Proteobacteria*, and *Actinobacteria*, which collectively form the functional backbone of the gut ecosystem and are recurrently altered in Crohn’s disease. Concentrated primarily in the colon, the gastrointestinal microbiota contributes to colonization resistance, short-chain fatty acid production, epithelial barrier maintenance, metabolic homeostasis, and continuous modulation of innate and adaptive immunity, thereby sustaining intestinal and systemic equilibrium [14,15,16,17].

**Figure 2:**
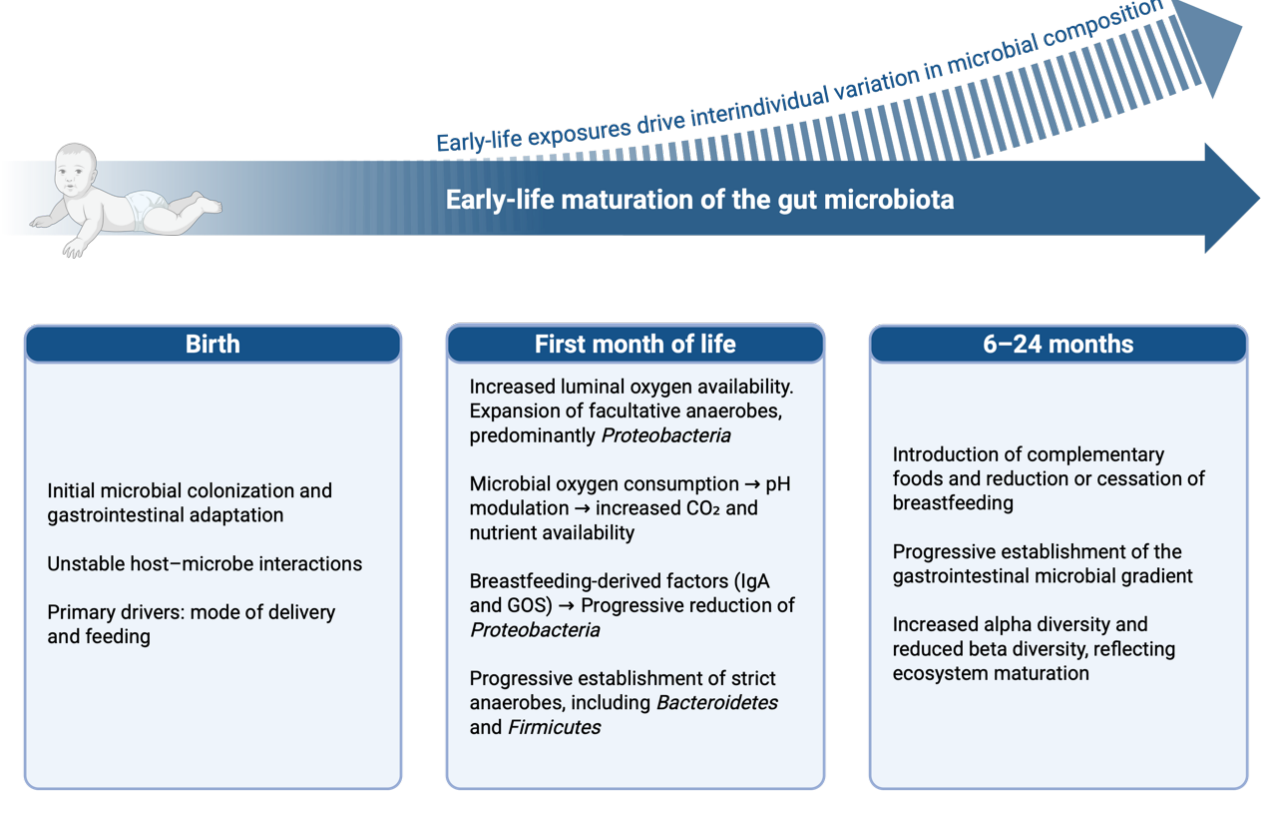
Early-life development of gut microbiota. Early-life microbial maturation follows a conserved developmental trajectory while giving rise to individualized microbial configurations shaped by early environmental exposures. This period represents a critical immunological window, during which microbiota–host interactions play a pivotal role in immune system programming, with long-term implications for immune tolerance and susceptibility to immune-mediated diseases. Abbreviations: CO_2_, carbon dioxide; IgA, immunoglobulin A; GOS, galactooligosaccharides. Created in BioRender. Saad, M. (2026) https://app.biorender.com/illustrations/695eac9bbc3a4a966b428c1d.

The establishment of this ecosystem follows a structured developmental trajectory shaped by early-life exposures. Colonization begins at birth through maternal and environmental microbial contact and remains highly dynamic during infancy, influenced by delivery mode, feeding practices, and the introduction of solid foods. In early life, the relatively oxygen-rich intestinal milieu favors facultative anaerobes, particularly *Proteobacteria* [14,16]. Progressive microbial oxygen consumption and luminal acidification are complemented by the establishment of critical host–microbe metabolic crosstalk. Specifically, as early colonizers begin to produce short-chain fatty acids (SCFAs), particularly butyrate, they fuel mitochondrial β-oxidation within colonocytes. This oxygen-intensive metabolic process maintains physiological epithelial hypoxia, which effectively limits oxygen diffusion into the lumen. This hypoxic state is the primary driver creating the permissive conditions for strict anaerobes, such as Bacteroidetes and Firmicutes, to dominate the mature gut community. Human milk components, including galactooligosaccharides (GOS) and secretory IgA, further shape microbial selection and immune tolerance (Figure 2). Perturbations during this developmental window have been associated with increased susceptibility to immune-mediated diseases [18,19].

Within this context, CD is consistently associated with reduced microbial diversity and a dysbiosis signature detectable across multiple gastrointestinal compartments [20]. Across cohorts, this pattern frequently includes enrichment of Enterobacteriaceae (along with *Pasteurellaceae*, *Veillonellaceae*, and *Fusobacteriaceae*) and depletion of Clostridiales and Bacteroidales taxa, supporting a recurrent disease-associated microbial configuration [21].

However, these compositional trends should not be interpreted as uniform disease markers. CD involves distinct anatomical segments in a patient-specific manner, and microbial alterations vary accordingly. In ileal CD, mucosa-associated communities commonly show increased *Escherichia coli* and reduced *Clostridiales* compared with controls and colonic-restricted disease, reinforcing the segment-dependent nature of dysbiosis. Importantly, microbial signatures differ across sampling compartments, and alterations in mucosal or deeper tissue layers may not be adequately captured in stool samples, limiting the interpretability of fecal-only profiling in segmental disease [22]. This distinction is critical because the resident mucosa-associated microbiota is much closer to the host’s immune cells and the epithelial barrier than the transient luminal flora. Consequently, findings are strongly influenced by both disease location and sampling site, substantially affecting the apparent dysbiosis signal, particularly in ileal CD, where dysbiosis signatures are often more pronounced in mucosal biopsies because they directly reflect localized inflammatory crosstalk [20,23].

Beyond anatomical compartmentalization, the reproducibility of microbial signatures is further challenged by the inherent heterogeneity of CD clinical presentations. Apparent dysbiosis signals are strongly influenced by disease location, sampling site, and inflammatory burden [24,25]. Associations between microbiota patterns and disease activity have been reported, including the ability to discriminate between active and remission states in some cohorts, yet taxon-level reproducibility remains inconsistent [25,26]. Heterogeneity across studies may reflect differences in disease stage, longitudinal fluctuation, prior surgical history, and therapeutic exposure, underscoring the need for compartment-aware and temporally structured designs [20,27].

Therapeutic variables further complicated the interpretation. Antibiotics can amplify dysbiosis signals, whereas immunosuppressive and biologic therapies may remodel mucosa-associated communities independently of intrinsic disease biology [20,28]. Taken together, dysbiosis in CD is best conceptualized not as a single compositional entity but as a context-dependent ecological state shaped by anatomical compartment, inflammatory activity, disease trajectory, and pharmacologic pressure.

## 5. Intestinal Microbiome and Physiological Functions

The intestinal microbiota contributes to a broad range of physiological processes essential for host homeostasis. In Crohn’s disease, disruption of these physiological functions does not occur uniformly but reflects segmental inflammation, transmural injury, and phenotype-specific ecological remodeling. For conceptual clarity, these functions can be grouped into protective, metabolic, and immunoregulatory roles, which are discussed in the following sections.

### 5.1. Protective Functions

The intestinal microbiota exerts critical protective functions that maintain mucosal homeostasis. One of its primary mechanisms is colonization resistance, whereby resident microbes occupy ecological niches along the intestinal surface, thereby limiting pathogen adhesion, invasion, and expansion while preserving epithelial integrity [19].

Through anaerobic fermentation of non-digestible substrates, especially dietary fibers, the microbiota produces short-chain fatty acids (SCFAs), including acetate, propionate, and butyrate. These metabolites serve as the primary energy source for colonocytes, reinforce epithelial barrier function, and exert anti-inflammatory and anticarcinogenic effects, partly by inhibiting histone deacetylases and modulating gene expression. Butyrate promotes colonic regulatory T-cell differentiation by inhibiting histone deacetylases, thereby supporting an anti-inflammatory immune balance in the gut [29]. In addition, SCFAs signal through G-protein–coupled receptors such as GPR43 (FFAR2), which regulates intestinal inflammatory responses and neutrophil recruitment [30], and GPR109A, which mediates the anti-inflammatory effects of butyrate and promotes IL-10–dependent regulatory pathways [17,30,31,32,33].

In CD, disruption of microbiota-mediated protective mechanisms has been linked to disease pathogenesis. Reduced abundance of *Faecalibacterium prausnitzii* in ileal mucosa has been associated with increased risk of postoperative endoscopic recurrence in CD [34]. Experimental data from the same study demonstrated anti-inflammatory properties of *F. prausnitzii* and its metabolites, including inhibition of NF-κB activation [34].

Furthermore, susceptibility to CD is strongly associated with NOD2 variants, supporting a role for impaired innate microbial sensing in disease development [35,36]. In ileal CD, reduced expression of Paneth cell α-defensins has been reported, indicating compromised antimicrobial defense in this phenotype [37]

In CD, therefore, protective failure reflects a multilayered disruption of host–microbial equilibrium, in which reduced metabolite signaling, defective innate sensing, and compromised epithelial antimicrobial defense converge to lower the threshold for persistent mucosal inflammation.

### 5.2. Metabolic Function of the Intestinal Microbiota

The human gastrointestinal tract digests approximately 85% of dietary carbohydrates and 66–95% of proteins and fats, whereas the remaining 10–30% reach the colon and are metabolized by the intestinal microbiota. The availability and composition of these substrates are significant determinants of microbial structure and metabolic output, directly influencing host energy balance, epithelial integrity, and immune tone [38,39,40,41]. The principal microbial metabolic pathways are outlined below according to nutrient class.

#### 5.2.1. Non-Absorbable Carbohydrates and Fibers

Non-digestible carbohydrates, including oligosaccharides such as fructooligosaccharides (FOS) and GOS, lignin, non-starch polysaccharides, and resistant starch, serve as key energy sources for gut microorganisms. In contrast to the human genome, which encodes a limited repertoire of glycan-degrading enzymes, the intestinal microbiota harbors an extensive array of carbohydrate-active enzymes (CAZymes). The CAZy database currently describes more than 130 families of glycoside hydrolases, 22 polysaccharide lyases, and 16 carbohydrate esterases, many of which are widely distributed among gut bacteria, particularly within the phyla *Bacteroidetes* and *Firmicutes* [42].

Microbial fermentation of dietary fibers generates a diverse set of metabolites, including gases (hydrogen, methane, and carbon dioxide), organic acids (lactate and succinate), alcohols (methanol and ethanol), and, most importantly, SCFAs. SCFAs, predominantly acetate, propionate, and butyrate, are absorbed in the colon and contribute up to 10% of total human caloric requirements. Their biological effects are complementary: acetate serves as an energy substrate for peripheral tissues and participates in hepatic lipogenesis and cholesterol synthesis; butyrate is the primary energy source for colonocytes, enhances epithelial barrier function, stimulates enteroendocrine secretion of leptin and glucagon-like peptide-1 (GLP-1), and mitigates the cytotoxic effects of bile acids and phenolic compounds; propionate is transported to the liver, where it contributes to lipid and cholesterol metabolism and can enter the Krebs cycle for peripheral oxidation [43,44,45,46,47].

In Crohn’s disease, depletion of SCFA-producing taxa such as *Faecalibacterium prausnitzii* and other Clostridiales is unlikely to represent an isolated metabolic defect. Instead, it reflects inflammation-driven ecological remodeling. Active mucosal inflammation increases epithelial oxygenation and nitrate availability, creating a metabolic environment that favors facultative anaerobes (e.g., Enterobacteriaceae) over obligatory anaerobes responsible for SCFAs production [20,25,48].

This oxygenation shift disrupts anaerobic fermentation networks, reducing butyrate availability and impairing epithelial AMPK activation, tight junction integrity, and regulatory T-cell differentiation [49,50,51]. In parallel, epithelial stress and barrier dysfunction further increase luminal oxygen diffusion, reinforcing facultative expansion and metabolic collapse, a self-amplifying ecological loop [25,52].

Importantly, multi-omic longitudinal studies demonstrate that even after clinical remission, functional microbial pathways often remain incompletely restored, suggesting persistent ecological instability rather than full metabolic recovery [25,52]. This bidirectional host–microbiome instability may partly explain why dysbiosis frequently persists despite endoscopic improvement.

#### 5.2.2. Proteins

Proteins that escape digestion in the upper gastrointestinal tract are metabolized by colonic bacteria, shaping microbial composition and generating a broad range of bioactive metabolites. High intake of animal protein, particularly red meat, favors the expansion of *Bacteroides* and *Clostridia* and the reduction of *Bifidobacterium*. In contrast to carbohydrate fermentation, protein fermentation is associated with the accumulation of potentially toxic metabolites that exacerbate mucosal inflammation and epithelial stress. Notably, the fermentation of sulfur-containing amino acids leads to the production of hydrogen sulfide (H2S). While endogenous H2S acts as a signaling molecule, its microbial overproduction can be toxic to colonocytes by inhibiting mitochondrial cytochrome c oxidase. This inhibition impairs the beta-oxidation of butyrate, triggering a vicious cycle of epithelial energy failure and compromising the mucosal barrier integrity. Crucially, a functional distinction must be made between such deleterious proteolytic pathways and the specific, beneficial metabolism of tryptophan. Unlike the toxic accumulation of ammonia and sulfides, tryptophan-derived indole and its derivatives act as essential ligands for the aryl hydrocarbon receptor (AhR), which is fundamental for maintaining epithelial barrier integrity and host–microbiome homeostasis [53,54].

Protein-derived microbial metabolites comprise diverse biochemical classes with neuroactive, inflammatory, and metabolic effects. High dietary protein-to-carbohydrate ratios shift microbial catabolism toward the production of neuroactive compounds that can influence host physiology via the gut–brain axis. In CD, the expansion of proteolytic bacteria, combined with low fiber intake, may increase the production of pro-inflammatory protein-derived metabolites, such as ammonia and H2S, while often diminishing the availability of protective tryptophan-derived indoles, thereby amplifying mucosal inflammation and systemic immune activation (Table 1) [55,56,57,58].

#### 5.2.3. Fats

High-fat diets induce profound changes in gut microbial composition and host metabolic and immune responses. Both experimental and clinical studies associate high-fat intake with dysbiosis, increased intestinal permeability, low-grade chronic inflammation, and enhanced adiposity, partly driven by bile acid pool remodeling. This environment favors the expansion of bile-acid–tolerant pathobionts, such as *Bilophila wadsworthia*, which has been linked to Th1-driven intestinal inflammation in experimental models of CD [59].

Adiposity-related microbial shifts further reflect this interaction, including reduced abundance of barrier-supporting taxa such as *Akkermansia muciniphila*. Importantly, the type of dietary fat exerts distinct selective pressures: saturated fats promote pro-inflammatory microbial configurations, whereas polyunsaturated fats generate alternative, potentially less inflammatory profiles. In parallel, lipid-derived microbial metabolites interact with short-chain fatty acids to regulate epithelial barrier integrity and immune tone, highlighting the integrated role of dietary fat and microbial metabolism in shaping intestinal inflammation (Table 1) [60].

Rather than detailing individual molecular pathways, Table 1 summarizes key microbial metabolites, their primary functional effects on the host, and the dominant microbial groups involved.

In CD, multi-omic profiling has shown that metabolic dysfunction is a core component of dysbiosis, including reduced abundance and activity of pathways involved in SCFAs production and other microbial metabolites relevant to epithelial homeostasis [61]. Longitudinal integrated analyses further demonstrate that active disease is accompanied by a reproducible shift in microbial functional programs, consistent with an inflammation-associated remodeling of community metabolism rather than purely taxonomic change. [25].

Metabolic disruption in CD also involves bile-acid handling, with IBD-associated dysbiosis linked to bile-acid dysmetabolism and altered bile-acid profiles that may influence intestinal inflammation through host–microbe feedback loops [62].

In addition, altered tryptophan metabolism is associated with inflammatory activity in IBD cohorts, including CD, supporting the concept that amino-acid–derived microbial/host metabolites represent another relevant metabolic layer in disease biology [63].

## 6. The Intestinal Mucus Layer

The integrity of the intestinal mucus barrier is essential for mucosal homeostasis. Reduced mucus production or increased degradation (due to congenital factors, limited substrate availability, or environmental exposures) predisposes to inflammation and is associated with conditions such as cancer and inflammatory bowel disease. Composed of mucins, antimicrobial peptides, and immunoglobulins, the mucus layer forms the primary physical and immunological barrier separating luminal microbes from the intestinal epithelium [64,65].

In the small intestine, the mucus barrier consists of a single, non-adherent layer that can be readily removed by aspiration [66]. Its detachment is microbiota-dependent, as germ-free mice require several weeks of bacterial colonization before normal mucus separation occurs. This process is partially mediated by the metalloprotease meprin β, which is induced by microbial colonization and cleaves MUC2 at the goblet cell surface, enabling mucus release into the lumen [67].

In the colon, the mucus barrier is more complexly organized. It is structured into an inner, dense layer that is largely impenetrable to bacteria and an outer, loose layer that can be colonized by commensals and used as a nutrient source. Secreted by goblet cells, the inner mucus can be subdivided into b1 and b2 layers, produced predominantly in the proximal and distal colon, respectively, with the latter remaining in proximity to the epithelium and in minimal contact with microbes [68,69].

Disruption of the mucus barrier alters microbial spatial organization and can promote colitis, underscoring its central role in both barrier integrity and immune tolerance. Under conditions of low intake of non-absorbable carbohydrates, particularly dietary fiber, the mucus layer itself may become a metabolic substrate. This shift favors the expansion of mucus-degrading bacteria, the thinning of the barrier, and increased susceptibility to mucosal inflammation [64,65,68,69].

**Table 1:** Bacterial metabolites and their beneficial effects on human health.

Metabolite	Involved Bacterial Agents	Production Pathway	Function
Butyrate [17,25,26,34,39,49,59,61,64,70,71,72,73,74,75,76,77,78,79,80,81,82,83]	*Clostridium* spp. *Faecalibacterium prausnitzii* *Coprococcus catus* *Anaerostipes hadrus*	Carbohydrate metabolism	**Mechanistic Pathways** HDAC inhibition Activation of GPR109A AMPK activation **Physiological Functions** Enhances intestinal barrier integrity (tight junctions) Modulate macrophage function Reduces colonic inflammation Increases insulin sensitivity **Alterations in CD** Reduced abundance of butyrate-producing taxa Reduced mucosal SCFAs availability
Propionate [17,25,26,39,59,61,64,74,75]	*Blautia obeum* *Coprococcus catus* *Roseburia inulinivorans* *Prevotella copri*	Carbohydrate metabolism	**Mechanistic Pathways** GPR41 activation SCFA–immune axis modulation **Physiological Functions** Reduces colonic inflammation Dampens innate immune responses to microbial stimuli Attenuates allergen-induced airway inflammation **Alterations in CD** Altered SCFAs profiles reported in active CD Reduced SCFA-producing communities in inflamed segments
Acetate [17,25,26,39,61,64,74,75]	Multiple SCFA-producing genera (e.g., *Prevotella*, *Bifidobacterium*)	Carbohydrate metabolism	**Mechanistic Pathways** GPR43 activation **Physiological Functions** Supports epithelial energy metabolism Promotes mucosal immune tolerance Contributes to SCFA-mediated metabolic effects **Alterations in CD** Shifted SCFAs balance in dysbiotic states
Indole [17,25,26,61,63,71,74,76,77,78,79]	*Lactobacillus* spp. *Bifidobacterium longum* *Bacteroides fragilis*	Tryptophan metabolism	**Mechanistic Pathways** AhR activation **Physiological Functions** Host–microbiota homeostasis via IL-22 Enhances intestinal barrier function Modulates host metabolic pathways. **Alterations in CD** Disrupted tryptophan metabolism observed in IBD cohorts Reduced indole derivatives linked to impaired AhR signaling
Indole-3-aldehyde [17,25,26,61,63,70,71,72,73,74,76,77,78,79]	*Lactobacillus* spp.	Tryptophan metabolism	**Mechanistic Pathways** AhR activation **Physiological Functions** Stimulates IL-22 production Maintains mucosal immune homeostasis **Alterations in CD** Altered tryptophan metabolism and reduced AhR pathway activity have been reported in IBD cohorts Potential impairment of IL-22–mediated epithelial repair
Indole-3-propionate [17,25,26,61,63,71,74,76,77,78,79]	*Clostridium sporogenes*	Tryptophan metabolism	**Mechanistic Pathways** Antioxidant activity AhR-related signaling **Physiological Functions** Protects against mucosal barrier injury Enhances epithelial homeostasis Alterations in CD * No direct human evidence in CD.
Conjugated linoleic acid (CLA) [17,25,26,59,61,63,72,73]	*Lactobacillus* spp.	Linoleic acid conversion	**Mechanistic Pathways** NF-κB suppression Lipid signaling modulation **Physiological Functions** Reduces inflammation Improves epithelial barrier structure Regulates lipid metabolism Alterations in CD * No direct human evidence in CD.
10-hydroxy-cis-12-octadecenoic acid [17,25,26,59,61,63,72,73]	*Lactobacillus* spp.	Linoleic acid derivative; lipid metabolism	**Mechanistic Pathways** NF-κB inactivation **Physiological Functions** Enhances intestinal barrier function Reduces mucosal inflammation Increases intestinal IgA production Alterations in CD * No direct human evidence in CD.

IL: Interleukin; IgA: Immunoglobulin A; SCFAs: Short-chain fatty acid; HDAC: Histone deacetylase; AhR: Aryl hydrocarbon receptor; AMPK: AMP-activated protein kinase; GPR41/GPR43: G-protein–coupled receptors 41 and 43; NF-κB: Nuclear factor kappa-light-chain-enhancer of activated B cells. * Limited human data. Table adapted and expanded from Gomaa EZ with additional supporting evidence [17,25,26,34,39,49,59,61,63,70,71,72,73,74,75,76,77,78,79,80,81,82,83].

In CD, mechanistic insights into mucus barrier dysfunction derive predominantly from studies of ileal disease, where impaired antimicrobial peptide expression, Paneth cell abnormalities, and altered epithelial–microbial interactions have been most consistently demonstrated. Evidence specific to purely colonic CD remains comparatively limited and heterogeneous. Reduced Paneth cell α-defensin expression has been demonstrated in ileal CD, supporting the notion that compromised antimicrobial defense is a phenotype-specific defect [37]. This reduction is more pronounced in patients carrying *NOD2* (CARD15) variants, linking impaired innate microbial sensing to diminished defensin expression [84]. In parallel, adherent-invasive *Escherichia coli* (AIEC) strains are enriched in ileal CD mucosa, where they adhere to epithelial cells and survive within macrophages, promoting sustained inflammatory signaling [10,85]. More broadly, in inflamed segments of CD, the structural organization of the mucus barrier is compromised; specifically, the ‘impenetrability’ of the inner mucus layer (the b2 layer in the distal colon) is frequently lost. This disruption represents more than a physical barrier failure; it is a critical immunological event that allows pathogen-associated molecular patterns (PAMPs) to access and activate Toll-like receptors (TLRs) on the apical surface of the epithelium in a disordered manner. This barrier breach facilitates the translocation of both commensal and pathogenic bacteria to the lamina propria, where they come into direct contact with the mucosal immune system. This increased mucosa-associated bacterial proximity to the epithelial surface supports the concept that inflammatory remodeling of the mucus interface facilitates persistent epithelial–microbial interaction and fuels chronic inflammation [86,87,88]

Current evidence suggests that mucus alterations in CD arise from the interaction of genetic susceptibility, impaired antimicrobial defense, and inflammation-driven remodeling of the epithelial barrier, rather than representing a uniform primary structural defect across all disease phenotypes.

## 7. Overview of Host–Microbiota Immune Crosstalk

The intestinal microbiota is an active regulator of the host immune system rather than a passive microbial community. By shaping both innate and adaptive immune pathways, commensal microorganisms provide essential signals for immune maturation, functional specialization, and long-term immune homeostasis [89,90].

A key interface in host–microbiota immune crosstalk is the intestinal barrier, particularly the mucus layer, which maintains spatial segregation between luminal microbes and the epithelium and integrates environmental signals into immune regulation [80,91]. This barrier is highly sensitive to dietary and environmental perturbations, and shifts in microbial metabolism, such as those induced by low-fiber intake, can compromise its integrity, promote mucus degradation, and increase susceptibility to intestinal inflammation [64,92].

Beyond its barrier-related effects, the microbiota actively instructs immune cell function. Signals derived from commensals modulate the activity of macrophages, dendritic cells, innate lymphoid cells, and direct adaptive immune differentiation into Th1, Th17, and regulatory T cell (Treg) lineages [89,93,94]. These effects are primarily mediated by microbial-derived metabolites, which serve as key molecular messengers in microbiota–immune crosstalk.

In addition to microbial metabolites, the intestinal microbiota provides essential signals for immune maturation and the maintenance of mucosal immune homeostasis [89]. Host antigen presentation pathways, including HLA-related mechanisms, contribute to Crohn’s disease susceptibility by modulating immune responses to microbiota-derived [95,96]. In Crohn’s disease, susceptibility genes involved in microbial sensing and bacterial handling, such as *NOD2*, support a model in which altered host responses to microbial stimuli contribute to disease pathogenesis [35]. Similarly, genetic variation in autophagy-related pathways (such as ATG16L1) has been associated with Crohn’s disease, reinforcing the link between host bacterial processing mechanisms and intestinal inflammation [97,98]. At the level of adaptive immunity, specific commensals can drive effector differentiation, as segmented filamentous bacteria induce intestinal Th17 cell responses [93]. Conversely, other members of the microbiota promote immune tolerance, as indigenous *Clostridium* species can induce colonic regulatory T cells [94]. Microbiota-derived short-chain fatty acids also contribute to regulatory immune tone by supporting colonic Treg homeostasis and differentiation [29,51]. Together, these findings support a framework in which disease emerges from disrupted host–microbiota immune homeostasis rather than from a single microbial trigger.

Short-chain fatty acids, particularly acetate, propionate, and butyrate, act as ligands for G protein–coupled receptors such as GPR41 and GPR43 and exert potent immunomodulatory effects. [29,51] Butyrate additionally functions as a histone deacetylase inhibitor, promoting Treg differentiation, enhancing epithelial tight-junction expression via AMPK activation, and reinforcing barrier integrity. Their depletion in CD compromises barrier function and regulatory immune pathways, thereby contributing to sustained inflammation (Table 1) [49,99,100].

Tryptophan-derived microbial metabolites constitute a key signaling axis that supports epithelial repair and mucosal homeostasis. Disruption of this pathway in CD impairs barrier maintenance and amplifies immune dysregulation (Table 1) [100,101,102,103].

**Figure 3:**
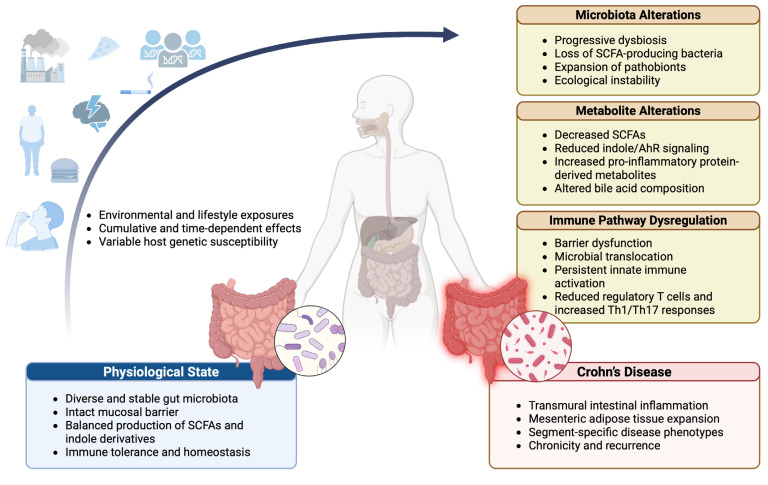
Integrated pathway linking gut microbiota, microbial metabolites, immune dysregulation, and Crohn’s disease. Under physiological conditions, a diverse and stable gut microbiota supports mucosal barrier integrity, appropriate production of SCFAs and indole derivatives, and immune tolerance. Cumulative environmental and lifestyle exposures, modulated by host genetic susceptibility, disrupt microbiome–host homeostasis, leading to progressive dysbiosis, altered microbial metabolism, and immune pathway dysregulation. These interconnected processes promote barrier dysfunction, microbial translocation, and sustained inflammatory responses, ultimately driving the development of Crohn’s disease, characterized by transmural inflammation, mesenteric adipose tissue expansion, segment-specific phenotypes, and chronic relapse. Abbreviations: AhR, aryl hydrocarbon receptor; SCFAs, short-chain fatty acids; Th, T helper cells; Treg, regulatory T cells. Created in BioRender. Saad, M. (2026) https://app.biorender.com/illustrations/695f9ae3a6cdc4f4796016c3.

Collectively, these mechanisms highlight the systemic reach of the intestinal microbiota. By releasing bioactive metabolites into the circulation, the gut microbiome influences immune responses at distant sites. Disruption of this structural–metabolic–immune axis establishes a permissive environment for dysbiosis and the chronic inflammatory phenotype characteristic of CD (Figure 3).

## 8. Correlation Between Microbiome and Crohn’s Disease

The human microbiome has co-evolved with its host through gradual selective pressures, fostering a finely tuned mutualism shaped by long-term environmental stability. When long-term host–microbiome adaptation is disrupted, the resulting imbalance creates a permissive environment for chronic immune-mediated diseases, including Crohn’s disease [104,105].

More detailed investigations into the gut microbiome of patients with CD began to emerge in the early 2000s. Although some variability exists at the phylum level, CD is consistently characterized by an expansion of pathogenic or pathobiont taxa and a depletion of beneficial commensals. Importantly, dysbiosis should not be viewed as a unidirectional trigger of disease. A central unresolved question remains whether dysbiosis represents an initiating ecological event in genetically susceptible hosts or an emergent property of chronic transmural inflammation. Current longitudinal data suggest that preclinical shifts occur years before diagnosis; however, these alterations are subtle and likely require host susceptibility and environmental reinforcement to translate into overt disease. Much of the current literature remains limited by cross-sectional design, stool-centric sampling, and insufficient control for disease location, inflammatory activity, and treatment exposure. These methodological constraints contribute to variability across studies and complicate causal interpretation of microbiome–disease associations in Crohn’s disease [25,106,107,108,109,110].

Microbiota changes are a dynamic component of CD pathogenesis: intestinal inflammation induces ecological remodeling characterized by increased epithelial oxygenation, expansion of facultative anaerobes, and loss of obligate SCFA-producing taxa. Although effective inflammatory control partially restores microbial composition, it rarely reestablishes a predisease equilibrium. Rather than a single pathogenic mechanism, CD arises from multilayered interactions among microbial ecology, host immunity, barrier integrity, and environmental exposures [25,106,107,108,109,110].

**Figure 4:**
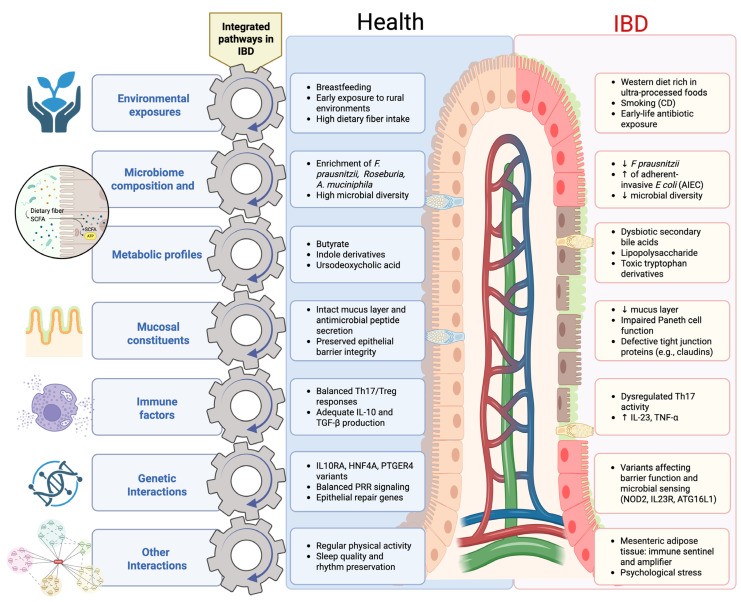
Integrated host–microbiome pathways in intestinal homeostasis and Crohn’s disease. Environmental exposures, microbiome composition, microbial metabolic outputs, mucosal barrier integrity, immune regulation, and host genetic factors interact to sustain intestinal homeostasis (**left**). In Crohn’s disease (**right**), disruption of these interconnected pathways results in dysbiosis, impaired barrier function, immune dysregulation, and chronic intestinal inflammation. The arrows within the gears illustrate the dynamic and interdependent nature of these pathways, indicating that coordinated movement across multiple domains—rather than an isolated alteration in a single factor—is required to drive either homeostasis or disease. Abbreviations: IBD, inflammatory bowel disease; CD, Crohn’s disease; SCFAs, short-chain fatty acids; Treg, regulatory T cells; Th, T helper cells; PRR, pattern-recognition receptors; AIEC, adherent–invasive *Escherichia coli*. Created in Created in BioRender. Saad, M. (2026) https://app.biorender.com/illustrations/695ebbd4754d686d88039e8a.

Interpreting the microbiome–CD relationship in a linear or reductionist manner leads to misleading conclusions. CD arises from interdependent biological networks involving host genetics, immune dysregulation, epithelial and vascular barrier dysfunction, microbial metabolites, environmental exposures, and microbial-derived toxins. Transmural inflammation, endothelial dysfunction, and impaired barrier integrity facilitate bacterial translocation or dissemination of microbial components to adjacent intestinal segments and extraintestinal sites, thereby sustaining chronic inflammation. Although the precise mechanisms governing microbial translocation remain incompletely defined, its role in disease propagation is increasingly recognized (Figure 4) [111,112].

An additional layer of complexity in Crohn’s disease lies in the spatial organization of host–microbiota interactions, which differs fundamentally from UC. Crohn’s disease is characterized by segmental and transmural inflammation, creating heterogeneous ecological niches across different intestinal segments and across the full thickness of the bowel wall [113]. These spatially distinct microenvironments shape local microbial composition, metabolic activity, and immune responses, contributing to marked inter- and intra-patient heterogeneity [25]. Multi-omics studies integrating mucosal, luminal, and tissue-level data have demonstrated that microbiome alterations in Crohn’s disease are strongly site-specific and influenced by local inflammatory and structural features [110]. This spatial heterogeneity helps explain variable therapeutic responses and the persistence of dysbiosis even after clinical or endoscopic control of inflammation [114].

Prospective studies provide further insights. A cohort of 3,483 healthy first-degree relatives of patients with CD revealed shifts in bacterial composition up to 5 years before diagnosis. Increases in mucin-degrading bacteria such as *Ruminococcus torques* and *Blautia*, accompanied by reductions in *Roseburia* (a genus associated with regulatory T-cell induction and IL-17 reduction), were observed in individuals who progressed to CD. In contrast, *Faecalibacterium prausnitzii,* known for its anti-inflammatory properties, declined years before disease onset. These findings highlight dysbiosis not only as a marker of disease but also as a potential early indicator of susceptibility [115,116,117].

An increased abundance of mucosa-associated bacteria with enhanced adhesive and invasive capacity further characterizes established CD. Despite extensive investigation, therapeutic manipulation of the microbiome in CD remains challenging. Probiotics and fecal microbiota transplantation (FMT) have demonstrated limited efficacy, whereas microbiome-targeted interventions have shown more consistent benefit in ulcerative colitis and pouchitis [118,119,120].

Genetic studies provide critical context for these observations. Despite large-scale genome-wide studies that have identified more than 200 susceptibility loci for inflammatory bowel disease, known genetic variants account for less than 15% of disease heritability. Prominent CD-associated genes, including *NOD2* and ATG16L1, are involved in microbial sensing, autophagy, and bacterial handling, supporting the view that CD arises from complex gene–environment–microbiome interactions rather than isolated genetic defects [35,97,98,121,122,123]. These interactions span multiple interconnected domains, including immune–endothelial signaling, environmental exposures (such as smoking, medications, and diet), microbial metabolic outputs, nutritional modulation, and proteomic alterations that may promote molecular mimicry or aberrant antigen presentation [124].

## 9. The Adipose–Microbiome Axis and Creeping Fat in CD

A hallmark of CD is transmural inflammation, which frequently leads to the development of “creeping fat” (CrF), the expansion of mesenteric adipose tissue encasing inflamed intestinal segments. Rather than a passive fat depot, CrF represents an active immunometabolic response to barrier disruption. Failure of metabolite-mediated signaling and structural compromise of the mucus layer permit selective translocation of bacteria or bacterial components into mesenteric compartments. Recent studies have identified *Clostridium innocuum* in the mesenteric fat of patients with CD. This bacterium acts as a ‘messenger’ of luminal dysbiosis to the extra-intestinal compartment, where it triggers a pro-inflammatory adipocytic response and activates profibrotic pathways through M2 macrophage polarization and stromal remodeling [98,99,100]. This translocation exemplifies the transmural nature of CD, where microbial signals cross the physical boundaries of the gut to remodel the surrounding adipose architecture and drive chronic inflammation [125,126,127].

In this context, mesenteric adipose tissue functions as a secondary immune barrier, attempting to contain microbial spread. However, this compensatory response often becomes maladaptive, as hypertrophied adipose tissue secretes pro-inflammatory mediators that further amplify intestinal inflammation, contributing to disease progression and segment-specific CD phenotypes [128].

## 10. Non-Bacterial Components of the Gut Microbiota on CD

Beyond bacteria, the CD-associated microbiome includes fungi, protozoa, bacteriophages, and archaea, each exerting distinct immunological and ecological effects [129]. Fungal dysbiosis, or mycobiome alteration, has emerged as a major contributor to chronic inflammation, with taxa such as *Candida* and *Malassezia* overrepresented, thereby driving immune activation and disease persistence [130,131]. Protozoa, including *Blastocystis hominis* and *Entamoeba histolytica*, are more frequently detected in CD and promote the secretion of pro-inflammatory cytokines, such as IL-1β, IL-6, and TNF-α [132,133].

The virome, composed mainly of bacteriophages, indirectly shapes mucosal immunity by modulating bacterial community structure and functional balance. In parallel, archaeal dysbiosis, notably reduced abundance of *Methanobrevibacter smithii*, has been associated with active disease, potentially through alterations in redox balance, expansion of facultative anaerobes, and bile acid dysregulation [134,135]. Collectively, these findings emphasize that a bacteria-centric view of CD is insufficient, as non-bacterial microbial constituents exert meaningful influence on disease pathophysiology.

These complex and interdependent pathogenic mechanisms help explain why direct manipulation of the microbiome in Crohn’s disease remains therapeutically challenging.

## 11. Microbiota Alterations According to Crohn’s Disease Phenotype

**Figure 5:**
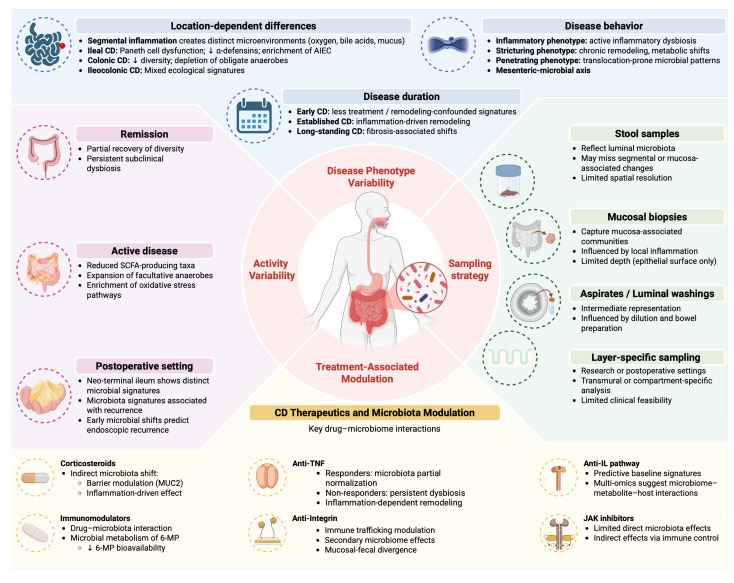
Context-Dependent Microbiota Variability in Crohn’s Disease. Microbiota signatures in CD are shaped by multiple, interacting sources of variability. Disease phenotype, inflammatory activity, and postoperative status generate distinct intestinal microenvironments that influence microbial composition and function. Therapeutic exposure further modulates microbiota profiles through both direct drug–microbe interactions and indirect inflammation-driven effects. In parallel, sampling strategy (stool, mucosal biopsy, luminal aspirates, or layer-specific analysis) critically determines the observed microbial signal, as each approach captures different ecological compartments. Together, these dimensions highlight that microbiota alterations in CD represent context-dependent ecological states rather than uniform disease signatures. Abbreviations: CD, Crohn’s disease; AIEC, adherent–invasive *Escherichia coli*; SCFAs, Short-chain fatty acids; MUC 2, Mucin 2; 6-MP, 6-mercaptopurine; TNF, Tumor necrosis factor; IL, Interleukin; JAK, Janus kinase. Created in BioRender. Saad, M. (2026) https://app.biorender.com/illustrations/69910d1a5fe1896151bda486.

Microbiota signatures in CD are not uniform; they vary by anatomical location and disease behavior. Segment-specific inflammation, tissue remodeling, and transmural involvement create distinct ecological niches that shape local microbial communities (Figure 5). Failure to stratify by phenotype risks conflating inflammation-driven dysbiosis with behavior-specific microbial patterns [136].

### 11.1. Location-Dependent Microbiota Differences

Location remains one of the most consistent modifiers of microbiota findings in CD. Ileal disease, particularly small-bowel–predominant CD, exhibits microbial features distinct from colonic involvement. In small intestinal CD, reduced diversity and altered relative abundances of specific taxa have been reported, although the magnitude of change varies across cohorts and sampling strategies [136]. Baseline microbiome configurations in small intestinal CD may cluster into biologically distinct groups that influence clinical response patterns, underscoring intra-phenotypic heterogeneity even within ileal disease [136].

Segmental inflammation generates microenvironments defined by bile acids, oxygen tension, mucosal permeability, and interactions with mesenteric fat, likely contributing to divergence between ileal and colonic microbial communities, although direct comparative datasets remain limited and methodologically heterogeneous [137,138,139]. Mucosa-associated sampling suggests enrichment of adherent bacteria and altered epithelial–microbial spatial relationships in ileal CD compared with colonic disease [140]. In contrast, stool-based analyses may fail to capture these segment-restricted alterations, highlighting the impact of sampling compartment on apparent location-specific signatures [141,142].

Although ileal CD has been more extensively characterized microbiologically, purely colonic CD remains underrepresented in high-resolution metagenomic studies, and direct head-to-head comparisons across locations are still sparse.

### 11.2. Microbiota Variation According to Disease Behavior

Beyond location, microbial patterns also vary with disease behavior, although inflammation itself remains a major confounder. The inflammatory phenotype is frequently associated with reduced alpha diversity, expansion of facultative anaerobes, and enrichment of inflammation-adapted taxa; however, many of these changes parallel inflammatory activity rather than representing stable behavior-specific signatures [143,144].

Fibrostenotic disease introduces additional complexity, as chronic remodeling, fibrosis, and altered motility may create ecological shifts distinct from purely inflammatory disease [145,146]. However, the interpretation of microbiota findings in stricturing CD is complicated by the fact that this phenotype frequently reflects longer disease duration and cumulative inflammatory burden. Moreover, prior bowel resection, common in fibrostenotic disease, can independently reshape baseline microbiome clustering in small intestinal CD, irrespective of current inflammatory activity. Fibrostenotic disease may involve distinct microbial–mesenchymal signaling pathways, although data remain limited [136]. Postoperative states, particularly in the neo-terminal ileum, constitute an additional ecological context with distinct microbial dynamics that may not reflect the original fibrostenotic process.

Penetrating luminal disease reflects transmural barrier disruption with increased potential for bacterial translocation. Although direct microbiota profiling in penetrating luminal CD remains limited, transmural inflammation likely alters the spatial distribution of microbes and mucosal adherence patterns. These changes may reflect altered tissue architecture rather than a distinct luminal compositional state.

Perianal CD, by contrast, represents a compartmentally distinct manifestation. Microbiota recovered directly from fistula tracts or drainage samples differ from fecal profiles, indicating that stool analysis does not adequately capture the microbial ecology of perianal disease [147,148,149]. Some datasets suggest associations between fistula-associated microbial composition and healing dynamics, although available studies remain small and heterogeneous [150,151].

Taken together, phenotype-associated variation in the microbiota likely reflects ecological adaptation to structural and immunological niches rather than stable microbial subtypes of Crohn’s disease. Distinguishing inflammation-driven remodeling from phenotype-specific ecology remains a critical unmet challenge.

## 12. Drug–Microbiota Interactions in Crohn’s Disease

Therapeutic exposure is a major confounder in microbiota studies of CD (Figure 5). In most cohorts, the intestinal ecosystem reflects not only disease biology but also pharmacologic pressure, complicating the attribution of microbial signatures to intrinsic disease mechanisms alone [152,153,154].

Corticosteroids can reshape microbial composition and influence mucin-related pathways in experimental systems, including modulation of MUC2 expression, yet available data suggest these effects are largely secondary to inflammation suppression rather than direct antimicrobial activity [155]. Thiopurines illustrate a more direct interaction: specific commensals can enzymatically convert 6-MP into inactive metabolites, reducing drug bioavailability and indicating that microbial metabolism may actively modulate pharmacologic exposure [156]

Biologic therapies, including anti-TNF agents and anti-integrin antibodies, are associated with partial restoration of microbial diversity and a reduction in pro-inflammatory taxa [157,158,159,160]. However, complete normalization of the microbiome is rarely achieved, even in patients who reach clinical or endoscopic remission. Whether the microbiome changes observed during biologic therapy represent direct drug–microbe interactions or are secondary to inflammation control remains unresolved. Disentangling primary ecological modulation from inflammation-driven remodeling will be essential for understanding whether therapeutic microbiome shifts have mechanistic or merely correlative significance [108].

Within the anti-IL pathway, baseline fecal microbiota configurations have been associated with responses to ustekinumab in CD, suggesting that pre-existing ecological states may influence therapeutic efficacy [161]. Integrated multi-omic analyses further indicate that microbiome–metabolite–host interactions may modulate treatment outcomes, pointing toward functional microbial states rather than purely compositional markers [162].

In contrast, data specifically addressing the impact of JAK inhibitors on the microbiota in CD remain scarce. Available in vivo and in vitro modeling suggests limited direct short-term effects on microbiota composition, with observed shifts likely mediated indirectly through immune suppression rather than intrinsic antimicrobial activity [152].

Collectively, available data indicate that therapies in CD function predominantly as modifiers of inflammation-dependent microbial states rather than primary ecological disruptors. As a result, microbiota profiles in treated patients often represent a composite of disease activity and therapeutic remodeling. Distinguishing predictive microbial biomarkers from secondary ecological consequences remains an unresolved challenge and requires rigorously controlled longitudinal designs.

## 13. Assessing the Microbiome

Interpretation of microbiome studies in Crohn’s disease requires careful consideration of disease location and sampling strategy, as methodological limitations substantially influence reported microbial signatures. Fecal microbiome analysis is the most commonly used approach, but its interpretation must account for disease location, as dysbiosis in CD is segment-specific. Alterations are more pronounced in ileocecal and ileocolonic disease, whereas in proximal CD (Montreal L1 or L4), fecal samples may be poorly representative due to the dominant signal from an unaffected colon. In these settings, mucosal biopsies or luminal aspirates provide a more reliable assessment of local microbial changes [20,24,163,164].

Amplicon-based techniques include 16S rRNA gene sequencing, widely used for bacterial profiling but offering limited functional and strain-level resolution, and Internal Transcribed Spacer (ITS) sequencing, which improves detection of non-bacterial components such as fungi and protozoa. Shotgun metagenomics enables simultaneous characterization of microbial composition, metabolic pathways, and virulence factors and has been pivotal in identifying functional signatures associated with CD. Long-read metagenomics further enhances resolution by enabling strain-level analysis, complete genome assembly, and detection of mobile genetic elements, thereby contributing to understanding microbial persistence and disease recurrence [20,114].

## 14. Microbiome-Targeted Therapies in Crohn’s Disease: Current Evidence, Limitations, and Future Directions

Despite strong mechanistic links between the gut microbiome and Crohn’s disease pathogenesis, translating these insights into effective therapies remains challenging. This gap reflects pronounced interindividual heterogeneity, segment-specific disease involvement, inflammation-driven microbial instability, and persistent host-derived ecological pressures [165,166]. Below, we summarize current microbiome-targeted therapeutic strategies and highlight emerging translational opportunities.

### 14.1. Dietary Modulation

Dietary interventions represent the most direct means of influencing gut microbial composition and function. Diets rich in fermentable fibers promote the production of SCFAs, supporting epithelial barrier integrity, regulatory immune pathways, and metabolic homeostasis. In contrast, Western dietary patterns (high in fat and animal protein) favor proteolytic fermentation, reduced SCFAs availability, expansion of mucus-degrading bacteria, and a pro-inflammatory milieu [167,168,169,170].

In CD, structured dietary protocols such as the Crohn’s Disease Exclusion Diet (CDED) aim to reduce exposure to pro-inflammatory dietary components while fostering a microbiome compatible with remission. Although effective in selected patients, diet remains an adjunctive therapy, with outcomes influenced by disease location, inflammatory burden, and baseline microbial composition [171,172,173].

### 14.2. Prebiotics and Postbiotics

Prebiotics (selectively utilized substrates) and postbiotics (microbial metabolites or structural components) exert immunomodulatory and barrier-enhancing effects by increasing SCFAs production, activating aryl hydrocarbon receptor (AhR) signaling, stimulating mucin secretion, and reinforcing epithelial resilience. Despite a strong mechanistic rationale, clinical evidence in CD is limited, and response variability reflects underlying microbial and inflammatory heterogeneity. Current data suggest greater potential when these agents are integrated into multimodal therapeutic strategies [174,175,176].

### 14.3. Fecal Microbiota Transplantation (FMT)

Although highly effective for recurrent *Clostridioides difficile* infection, FMT has shown limited efficacy in controlled trials of CD. This likely reflects the transmural inflammation characteristic of CD, reduced microbial resilience, and impaired engraftment in the setting of ongoing epithelial and immune dysfunction. Consequently, FMT is not currently recommended as a routine therapeutic option on CD [177,178].

### 14.4. Live Biotherapeutic Products (LBPs)

Live Biotherapeutic Products (pharmaceutical-grade microorganisms with defined therapeutic intent) represent an emerging class of microbiome-based therapies. Candidates such as *Faecalibacterium prausnitzii*, consistently associated with anti-inflammatory effects and sustained remission, are of particular interest. However, challenges, including oxygen sensitivity, impaired colonization under inflammatory conditions, and host-specific ecological constraints, limit its current applicability. LBPs may be most effective when combined with dietary interventions or biologic therapies within a precision medicine framework [48,179].

### 14.5. Future Therapeutic Directions

Several emerging strategies promise to advance microbiome-based therapies in CD:

- Multi-omics integration: Combining metagenomics, metabolomics, transcriptomics, and proteomics to identify strain-level signatures and patient-specific therapeutic targets [180,181,182].
- Microbial metabolites as therapeutics: Development of SCFAs, indole derivatives, and lipid-derived metabolites as postbiotic drugs, bypassing colonization challenges [183].
- Bacteriophage therapy: Strain-specific targeting of pro-inflammatory bacteria to modulate dysbiosis with high precision [181,184].
- Microbiome-guided stratification: Use of microbial profiles as biomarkers to predict treatment response, disease course, and postoperative recurrence [95,159,184].

Collectively, these limitations highlight critical gaps in our current understanding of host–microbiome interactions in Crohn’s disease.

## 15. Knowledge Gaps and Future Perspectives

Despite significant advances in microbiome research, key gaps still limit its clinical translation in CD. There is no consensus definition of dysbiosis, and reported microbial signatures vary substantially across disease locations, inflammatory activity, and analytical platforms, hindering cross-study comparisons and biomarker development [25]. In addition, establishing causality remains challenging, as microbial alterations detected in early or preclinical CD are strongly influenced by inflammation, diet, and therapeutic exposures [25].

Most studies remain predominantly bacteria-centered, leaving fungi, viruses, protozoa, and archaea underexplored despite growing evidence of their immunological and ecological relevance in CD [130]. Variability in sampling strategies, sequencing depth, and bioinformatic pipelines further limits reproducibility and standardization across cohorts [52,159].

Although microbiome profiling shows promise for predicting therapeutic response and postoperative recurrence, these approaches remain exploratory, and no validated microbiome-based stratification tools are currently available for routine clinical use [52,159]. Bridging this translational gap will require harmonized methodologies, standardized reporting frameworks, and prospective validation in well-phenotyped cohorts, ideally integrating microbial composition with functional metabolic readouts and clinical phenotypes.

Future progress will depend on longitudinal, mechanism-oriented designs with strain-level resolution, integration of multi-omics datasets, and ecological modeling capable of disentangling causal host–microbiome interactions. Advancing from descriptive associations toward functionally informed, clinically actionable models may ultimately enable microbiome-guided precision strategies in Crohn’s disease.

## 16. Conclusions

The gut microbiota is a fundamental component of intestinal physiology, contributing to metabolic balance, barrier integrity, and immune regulation. Alterations in microbial composition and function accumulate over time due to genetic susceptibility, environmental exposures, diet, immune activation, and therapeutic remodeling. In CD, these ecological perturbations are not confined to disease initiation but actively shape progression, complications, transmural inflammation, and segment-specific phenotypes.

Despite major advances enabled by high-resolution sequencing and multi-omics technologies, the intestinal microbiome and its layered interactions with host pathways remain incompletely resolved. Current evidence supports a bidirectional model in which dysbiosis is both a driver and a consequence of chronic inflammation, which in turn reinforces immune activation, barrier dysfunction, microbial translocation, extraintestinal manifestations, and mesenteric adipose expansion.

The spatial, phenotypic, and therapeutic heterogeneity of Crohn’s disease requires microbiome research to move beyond stool-based compositional profiling toward compartment-aware, longitudinal, and strain-resolved models. Only by integrating disease location, behavior, inflammatory state, and treatment exposure can microbiome science meaningfully inform disease stratification and precision medicine in CD.

Together, these perspectives reposition dysbiosis as a dynamic ecological process embedded within host structure and immune biology rather than a static taxonomic imbalance. Advancing this integrative, compartment-aware framework will be critical to transforming microbiome research in Crohn’s disease from descriptive profiling into mechanistically informed precision medicine.

## Data Availability

No new data were created or analyzed in this study. Data sharing is not applicable to this article.

## Abbreviations

The following abbreviations are used in this manuscript:

AhR	Aryl hydrocarbon receptor
AIEC	Adherent–invasive *Escherichia coli*
AMP	Antimicrobial peptides
AMPK	AMP-activated protein kinase
CAZymes	Carbohydrate-active enzymes
CD	Crohn’s disease
CDED	Crohn’s Disease Exclusion Diet
CLA	Conjugated linoleic acid
CO2	Carbon dioxide
CrF	Creeping fat
FMT	Fecal microbiota transplantation
FFAR2	Free fatty acid receptor 2
FOS	Fructooligosaccharides
GLP-1	Glucagon-like peptide-1
GOS	Galactooligosaccharides
GPR41	G-protein–coupled receptor 41
GPR43	G-protein–coupled receptor 43
GPR109A	G-protein–coupled receptor 109A
HDAC	Histone deacetylase
IBD	Inflammatory bowel disease
IgA	Immunoglobulin A
IL	Interleukin
ITS	Internal transcribed spacer
LBPs	Live biotherapeutic products
M2	Macrophage polarization
MeSH	Medical Subject Headings
MUC2	Mucin 2
NF-κB	Nuclear factor kappa-light-chain-enhancer of activated B cells
PRR	Pattern-recognition receptors
SCFAs	Short-chain fatty acids
Th	T helper cells
Treg	Regulatory T cells
UC	Ulcerative colitis
